# Research on Precise Attitude Measurement Technology for Satellite Extension Booms Based on the Star Tracker

**DOI:** 10.3390/s24206671

**Published:** 2024-10-16

**Authors:** Peng Sang, Wenbo Liu, Yang Cao, Hongbo Xue, Baoquan Li

**Affiliations:** 1 Laboratory of Advanced Measurement Technology, National Space Science Center, Chinese Academy of Sciences, Beijing 100190, China; sangp@nssc.ac.cn (P.S.); liuwenbo21@mails.ucas.ac.cn (W.L.); caoyang@nssc.ac.cn (Y.C.); 2 University of Chinese Academy of Sciences, Beijing 100049, China; 3 State Key Laboratory of Space Weather, National Space Science Center, Chinese Academy of Sciences, Beijing 100190, China; hbxue@nssc.ac.cn

**Keywords:** space extension boom, attitude measurement, star tracker, magnetic field measurement

## Abstract

This paper reports the successful application of a self-developed, miniaturized, low-power nano-star tracker for precise attitude measurement of a 5-m-long satellite extension boom. Such extension booms are widely used in space science missions to extend and support payloads like magnetometers. The nano-star tracker, based on a CMOS image sensor, weighs 150 g (including the baffle), has a total power consumption of approximately 0.85 W, and achieves a pointing accuracy of about 5 arcseconds. It is paired with a low-cost, commercial lens and utilizes automated calibration techniques for measurement correction of the collected data. This system has been successfully applied to the precise attitude measurement of the 5-m magnetometer boom on the Chinese Advanced Space Technology Demonstration Satellite (SATech-01). Analysis of the in-orbit measurement data shows that within shadowed regions, the extension boom remains stable relative to the satellite, with a standard deviation of 30′′ (1σ). The average Euler angles for the “X-Y-Z” rotation sequence from the extension boom to the satellite are [−89.49°, 0.08°, 90.11°]. In the transition zone from shadow to sunlight, influenced by vibrations and thermal factors during satellite attitude adjustments, the maximum angular fluctuation of the extension boom relative to the satellite is approximately ±2°. These data and the accuracy of the measurements can effectively correct magnetic field vector measurements.

## 1. Introduction

In modern spacecraft design and mission planning, the attitude measurement of long, deployable, lightweight, low-deformation, and high-stowage-efficiency space structures is a critical task [1,2]. Precise attitude measurement and control are key factors in ensuring mission success. In particular, for the precise measurement of magnetic field vectors, satellite-based magnetometry has emerged as an effective means for global geomagnetic field detection in recent years, with applications in Earth science, space science, geomagnetic navigation, seismology research, and other related fields [3,4]. However, due to the inherent residual magnetism of the satellite itself, which can cause significant interference with the in situ measurement of the spatial magnetic field, it is necessary to employ an extended structure to position the magnetometer’s magnetic field sensing probes away from the main body [5,6]. This strategy aims to minimize the interference of the residual magnetism of the main body on the accurate measurement of the magnetic field.

In the space orbital environment, factors such as temperature fluctuations, gravitational variations, and mechanical vibrations can lead to the deformation of deployable structures following their installation [7]. This can alter the reference coordinate system of the magnetometer, severely affecting the accuracy of magnetic field vector measurements [8]. Therefore, it is essential to precisely and continuously monitor the posture changes of the deployable structure that holds the magnetometer probe in order to correct the relationship between the vector magnetic field and the original reference coordinate system [9]. The required attitude measurement precision for this task is at the arcsecond level, covering multiple degrees of freedom, including the extension boom’s yaw, pitch, and roll movements [10].

In February 2000, during NASA’s Shuttle Radar Topography Mission (SRTM), an Attitude and Orbit Determination Avionics (AODA) system was used to achieve precise determination of the length and end-point orientation of a 60-m deployable structure as well as the position and orientation of the shuttle in the Earth-centered inertial coordinate system. The AODA system consisted of a GPS receiver system, an electronic distance meter (EDM), an Attitude Beacon Tracker (ATT), star trackers (STA), an Inertial Reference Unit (IRU), and the AODA Sensor Panel (ASP). This complex sensor combination achieved an estimation accuracy of 2 mm for the interferometric baseline length, 9 arcseconds for attitude, and 1 mm for position, meeting the mission requirements of SRTM [11].

In September 2012, the Nuclear Spectroscopic Telescope Array (NuSTAR) carried two side-by-side grazing-incidence X-ray optics with a focal plane consisting of two CdZnTe (Cadmium Zinc Telluride) crystal X-ray detectors [12]. A 10.9-m deployable mast connected the X-ray optics to the focal plane, and a star tracker module and metrology laser-detection module were used to monitor the attitude of the deployable structure. The quaternion attitude information obtained from the star tracker provided insights into the deviation of the X-ray optical axis, while the metrology laser-detection module was used to calculate the displacement of the optical system relative to the focal plane and the twisting angle of the optics around the deployable mast.

In November 2013, the European Space Agency (ESA) successfully launched the Swarm constellation, a geomagnetic low Earth orbit (LEO) mission [13]. Each of the three geomagnetic monitoring satellites was equipped with a 4-m deployable boom. A Vector Field Magnetometer (VFM) and three star trackers were installed at the 2-m position on the boom. The optical axes of the three star trackers were positioned at 90° angles to each other, ensuring that at any given time, no more than one star tracker would be affected by sunlight or Earth’s reflected light. The star tracker unit was equipped with a stray light suppression system to minimize the impact of thermal gradients caused by solar radiation on the optical system. The measurement accuracy of a single star tracker was about 2 arcseconds along the viewing axis but larger, at 46 arcseconds, in the roll direction.

Considering various attitude measurement technologies for deployable structures, the use of star trackers offers relatively higher measurement accuracy and stability, as shown in Table 1. The advantages of star trackers are particularly pronounced in deep space exploration, high-orbit missions, and long-duration missions. They provide comprehensive and reliable attitude information, are easy to process, and require minimal resources, making them an ideal choice for high-precision attitude measurement.

A star tracker is an instrument that measures attitude by referencing stars [19]. Since stars have very small angular diameters, typically in the milliarcsecond range, and their right ascension and declination are precisely known, star trackers offer high absolute attitude measurement accuracy without cumulative errors over time or distance [20]. However, star trackers that can be used directly for precise attitude measurement of extension booms generally face challenges related to their size, weight, power consumption, and significant residual magnetism. In recent years, with the development and advancement of CMOS active pixel sensor technology, CMOS image sensors have emerged as a new trend in star tracker applications. This is due to their simple driving sequence, low power consumption, and high integration, making CMOS image sensors an increasingly attractive option for star tracker development [21,22].

In the past, in China’s spaceborne magnetic field vector measurement field, there was a lack of effective methods to measure the attitude changes of deployable structures, and the impact of these changes on magnetic field vector measurements was largely neglected. However, as the precision of magnetic field measurements continues to improve, the influence of deployable structure attitude changes on measurement accuracy can no longer be ignored. This necessitates high-precision, real-time in-orbit attitude measurement technology for deployable structures to correct the deformation’s impact on vector magnetic field measurements and enhance data reliability and accuracy.

On 27 July 2022, the Chinese Advanced Space Technology Demonstration Satellite (SATech-01) was successfully launched into sun-synchronous orbit (SSO) [6]. Among the scientific instruments aboard was a high-precision geomagnetic measurement system, consisting of a CPT magnetometer equipped with micro-sensors, a small anisotropic magnetoresistance magnetometer (AMRM), and a nanosatellite star tracker (NST). These components were integrated on a stable platform designed to simultaneously measure both scalar and vector components of the magnetic field as well as the satellite’s attitude [23]. Furthermore, on November 7 of the same year, with the successful deployment of a non-magnetic telescopic tubular mast (TTM), the payload system officially entered its operational phase. During this process, the integrated vector magnetic field and attitude detection technology of the AMRM and NST underwent in-orbit testing and validation, confirming that their performance met the expected design standards.

## 2. Materials and Methods

**Figure 1:**
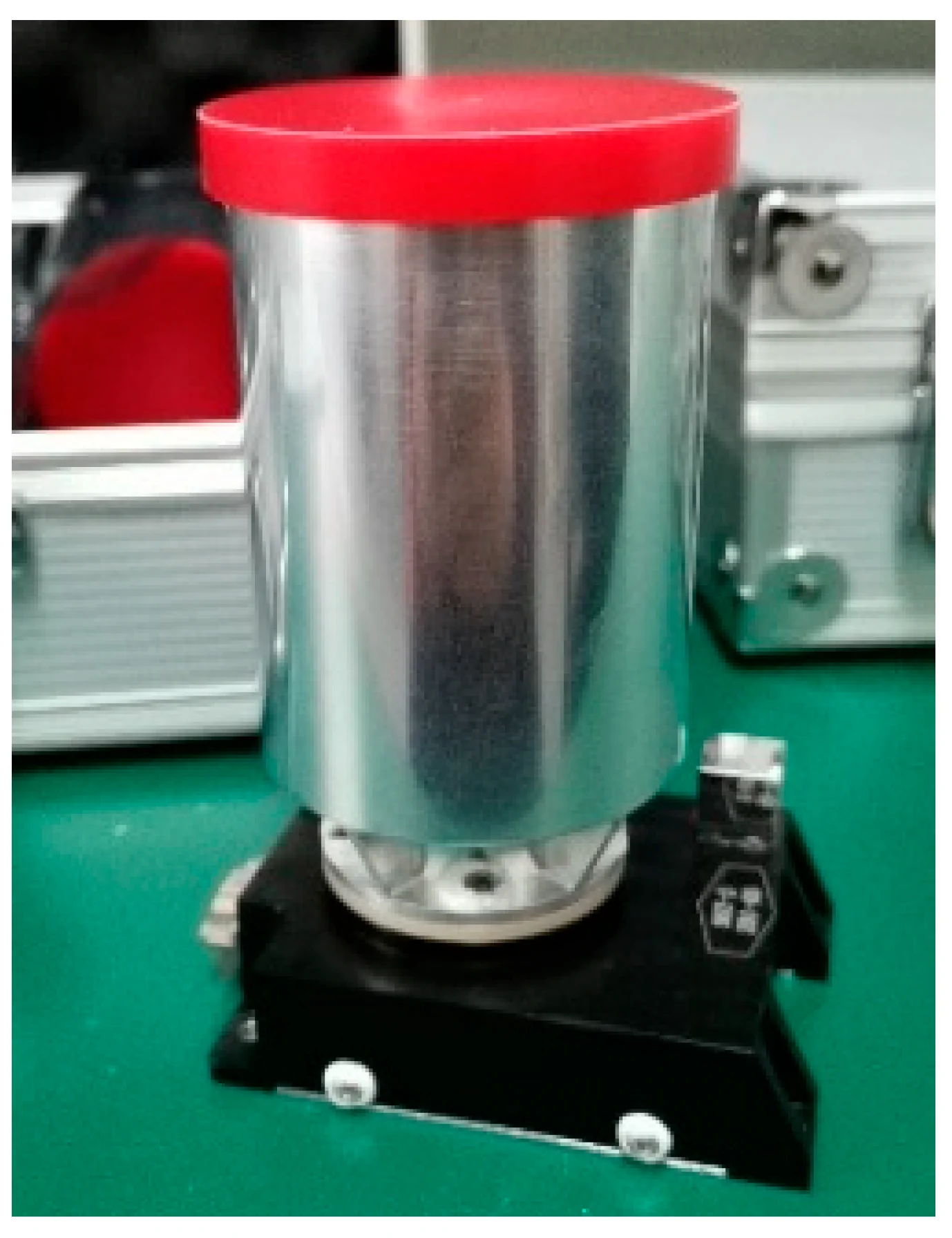
Self-developed nano-star tracker.

Considering the precise attitude measurement requirements and technical characteristics of extension booms, the star tracker used in boom attitude measurement systems must not only be capable of accurate and rapid attitude measurements but also meet the demands for low power consumption and miniaturization. This paper presents the development of a nano-star tracker based on a CMOS image sensor, with a unit weight of 150 g (including the baffle), and a prototype was successfully completed, as shown in Figure 1. In addition to its application in the attitude measurement of magnetometer booms, this nano-star tracker was previously employed by several commercial space companies for satellite attitude measurement and has a proven track record of multiple successful in-orbit missions.

Star trackers are currently the most accurate devices for space attitude measurement, and measurement accuracy is the most critical metric for evaluating their performance. The final measurement accuracy of a star tracker depends on the accuracy of single-star measurements, which are primarily influenced by factors such as pixel resolution, centroid location error, and calibration error. This accuracy can be estimated using the following equation [24]:

(1) $$\sigma_{gle\_star}=\frac{A_{FOV}}{N_{pixel}}\sigma_{centroid},$$

where $A_{FOV}$ represents the star tracker’s field of view; $N_{pixel}$ is the number of pixels along one edge of the image sensor; and $\sigma_{centroid}$ is the centroid location accuracy. With the estimation formula for single-star measurement accuracy established, we can further derive the formula for estimating the attitude measurement accuracy of the star tracker:

(2) $$\sigma_{cross-boresight}=\frac{A_{FOV}}{N_{pixel}}\frac{\sigma_{centroid}}{\sqrt{N_{star}}},$$

(3) $$\sigma_{roll}=\mathrm{atan}\left(\frac{\sigma_{centroid}}{0.3825N_{pixel}}\right)\frac{1}{\sqrt{N_{star}}}.$$

In this context, $\sigma_{cross-boresight}$ represents the pointing accuracy of the star tracker; $\sigma_{roll}$ denotes the roll accuracy; and $N_{star}$ is the number of observed stars. For a two-dimensional N × N pixel focal plane, the average distance from the star to the center of the focal plane is 0.3825 [24]. From these two equations, it can be seen that in addition to the effect of centroid positioning accuracy on the precision of the star tracker, higher pixel resolution ( $\frac{A_{FOV}}{N_{pixel}}$ ) leads to greater star tracker accuracy ( $\sigma_{cross-boresight}$ ). Additionally, the more stars the star tracker identifies and observes ( $N_{star}$ ), the higher its accuracy ( $\sigma_{roll}$ ). However, when the pixel resolution increases without a corresponding increase in the number of pixels, the field of view of the star tracker decreases. Similarly, to increase the number of identified stars while maintaining the same detection capability, the star tracker’s field of view must be expanded. Therefore, improving the attitude measurement accuracy of the star tracker by increasing pixel resolution or the number of stars observed presents a trade-off.

After comprehensive consideration, the EV76C560 chip from TELEDYNE e2v (Grenoble, France) was selected as the image sensor. This chip features a pixel size of approximately 5.3 µm, a pixel array of 1280 × 1024, and a power consumption of 200 mW. This choice balances high resolution with excellent pixel sensitivity, and its low power consumption meets the operational requirements. Additionally, the EV76C560 is compatible with the Global Shutter mode, which eliminates the need to account for rolling shutter distortion during image processing [25]. This feature is particularly important for long-exposure applications in star trackers as it significantly improves star map matching rates and enhances attitude measurement accuracy. On the data communication side, the EV76C560 supports image transmission in an 8-bit or 10-bit parallel synchronous transfer interface, with register configuration controlled via the Serial Peripheral Interface (SPI) bus. Both of these interface circuits are well-suited to ARM processors.

**Figure 2:**
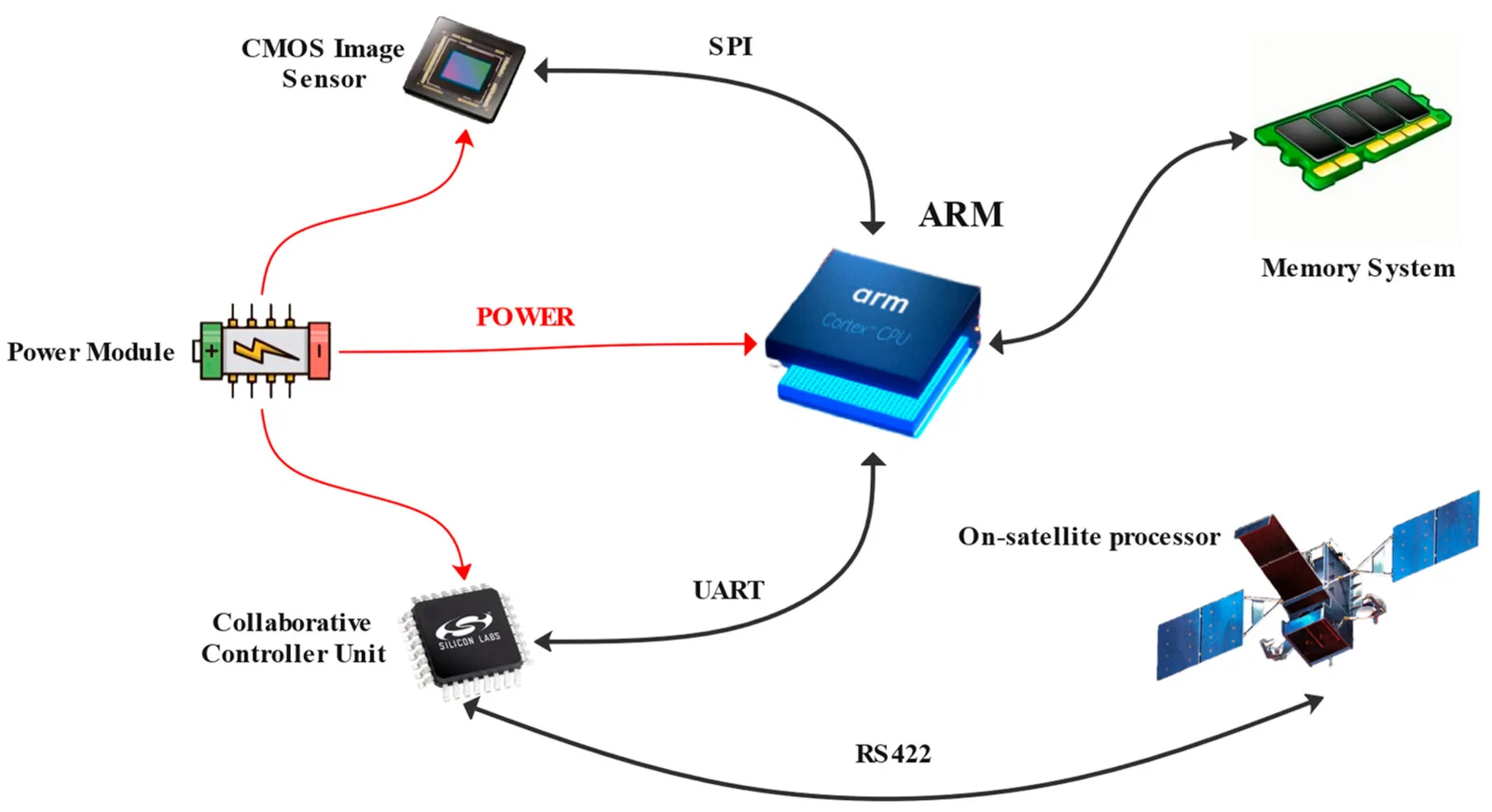
Electronic functional block diagram of the star tracker.

To meet the requirements of low power consumption and miniaturization in the attitude measurement system for extension booms, the nano-star tracker developed in this paper employed a CMOS + ARM architecture, which is resource-efficient and low-power. Additionally, to satisfy the reliability standards required for space applications, a high-grade, low-power microcontroller was integrated as a coprocessor to handle system management. Figure 2 shows the electronic functional block diagram of the star tracker.

**Figure 3:**
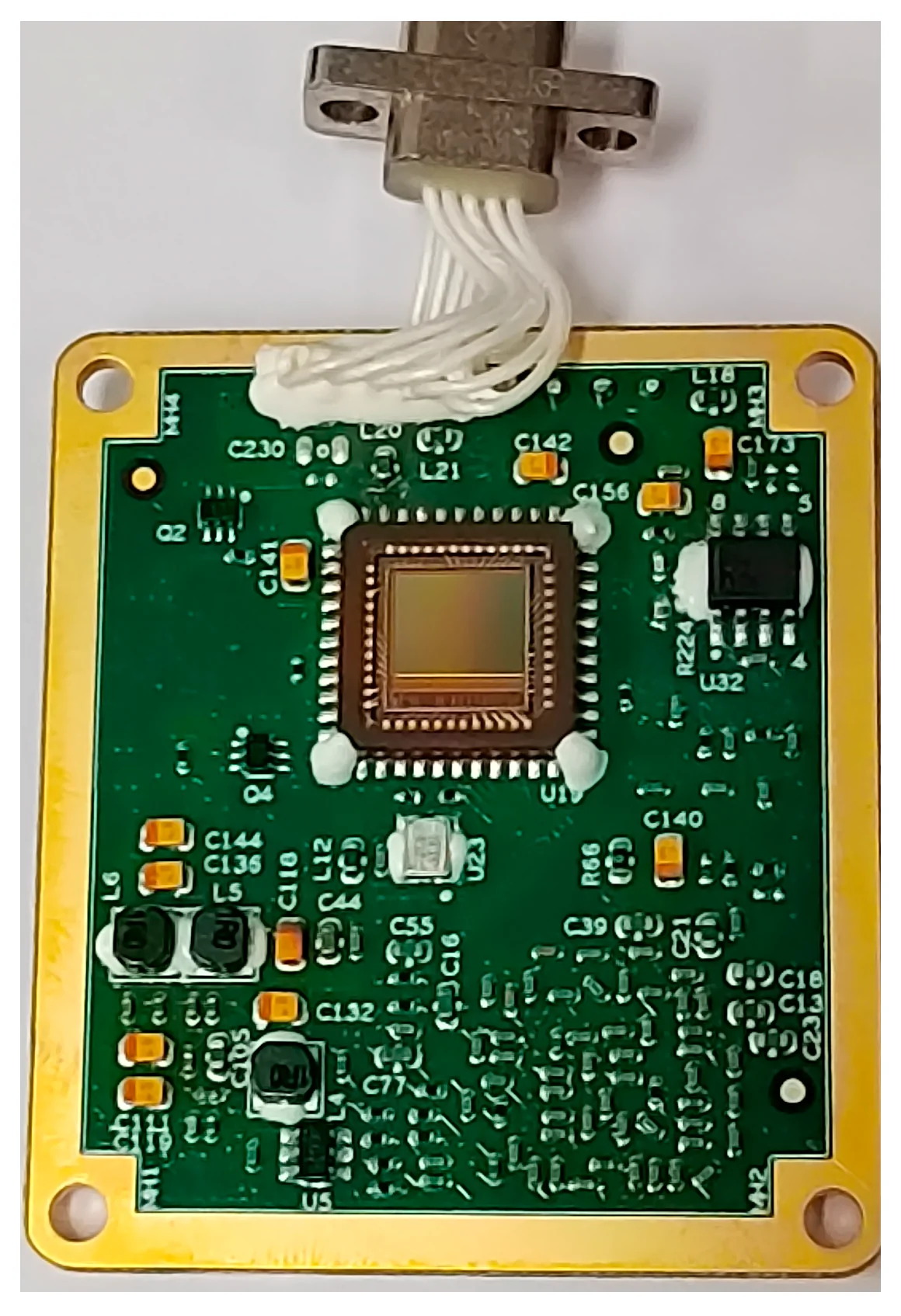
Photograph of a star tracker circuit board.

To achieve the miniaturization of the star tracker and to accommodate the focal length adjustment needed for the defocused optical system, a single-board design was adopted in the electronics design. Figure 3 shows the actual photo of the circuit board after fabrication was completed. During the design process, unnecessary components for debugging were removed, and other necessary interfaces, such as those required for testing, were extended through Flexible Printed Circuit (FPC) connections.

In the software design of the nano-star tracker, three operational modes were considered—Boot Load Mode, Working Mode, and Optimization Mode. The specific descriptions are as follows:

- Boot Load Mode: Upon powering on, the star tracker enters this mode for a certain period. In this mode, the coprocessor functions normally, providing standard com-munication capabilities. The ARM main processor performs a self-check during startup and receives operating parameters injected by the coprocessor. Meanwhile, the CMOS image sensor completes its initialization;
- Working Mode: After remaining in Boot Load Mode for some time and completing the self-check, the ARM processor transitions the star tracker into Working Mode, which is the tracker’s default operational mode. In this mode, the CMOS image sensor performs continuous exposures at a frequency of 5 Hz. The ARM processor timestamps each image frame precisely, executes star recognition, centroid localization, star map matching, and attitude computation algorithms. The coprocessor updates the latest attitude quaternion data and other engineering parameters in the cache. Upon receiving a telemetry polling command from the host computer, it transmits the most recent attitude quaternion;
- Optimization Mode: In this mode, the star tracker halts exposure and computation and the CMOS image sensor enters standby. The coprocessor can control the ARM processor to perform various tasks such as self-checks, injecting operational parameters, and capturing star image data based on different communication commands. The star tracker can switch between Optimization Mode and Working Mode via communication commands.

The default exposure time for the nano-star tracker was set to 100 ms (adjustable as needed). During full operation in orbit, the star pattern recognition utilizes the Lost In Space (LIS) mode rather than the traditional tracking recognition mode commonly used in star trackers. By optimizing the software architecture, the combined time for each frame’s exposure, star pattern matching, and attitude calculation is less than 120 ms. This allows the star tracker to achieve an attitude update rate of 5 to 8 Hz in the LIS mode, which meets the attitude control requirements of the extension boom.

**Figure 4:**
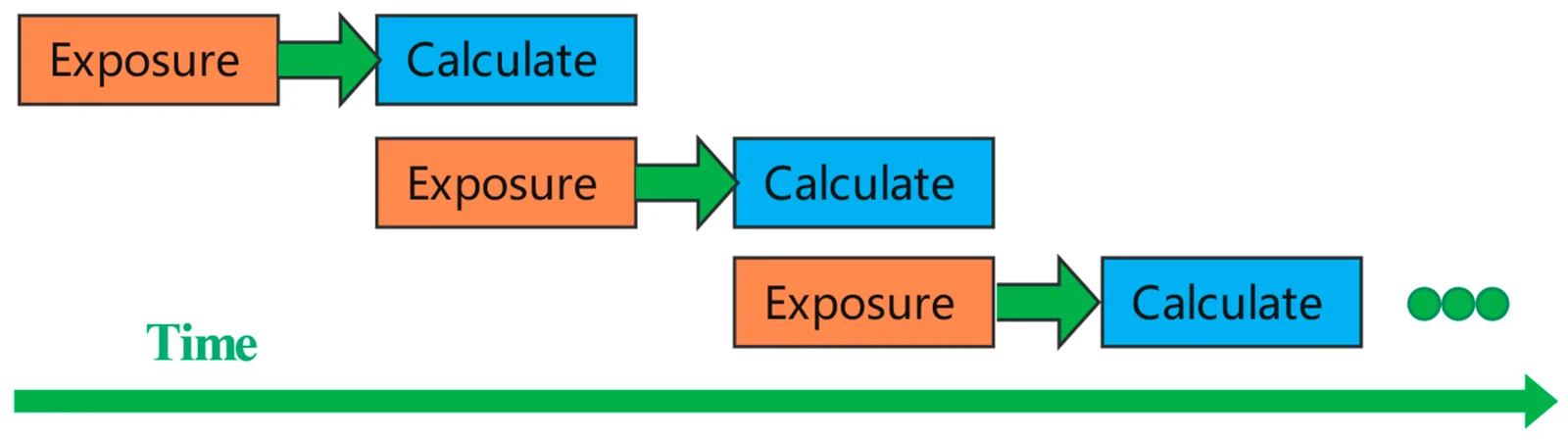
Schematic diagram of the multi-tasking pipeline of the star tracker.

To ensure low power consumption, a more powerful but higher power-consuming processor was not chosen. Instead, a multi-tasking pipeline model was designed, as illustrated in Figure 4. In this pipeline, star pattern acquisition and algorithmic computations can be performed in parallel. During the CMOS exposure time, the software can carry out operations such as star point extraction, star pattern matching, and attitude calculation for the previous frame. This design ensures that the attitude update rate only depends on the CMOS exposure and transmission times. Given a maximum exposure time of 100 ms and an image transmission time of less than 20 ms, the system meets the single-cycle requirement of 120 ms.

**Figure 5:**
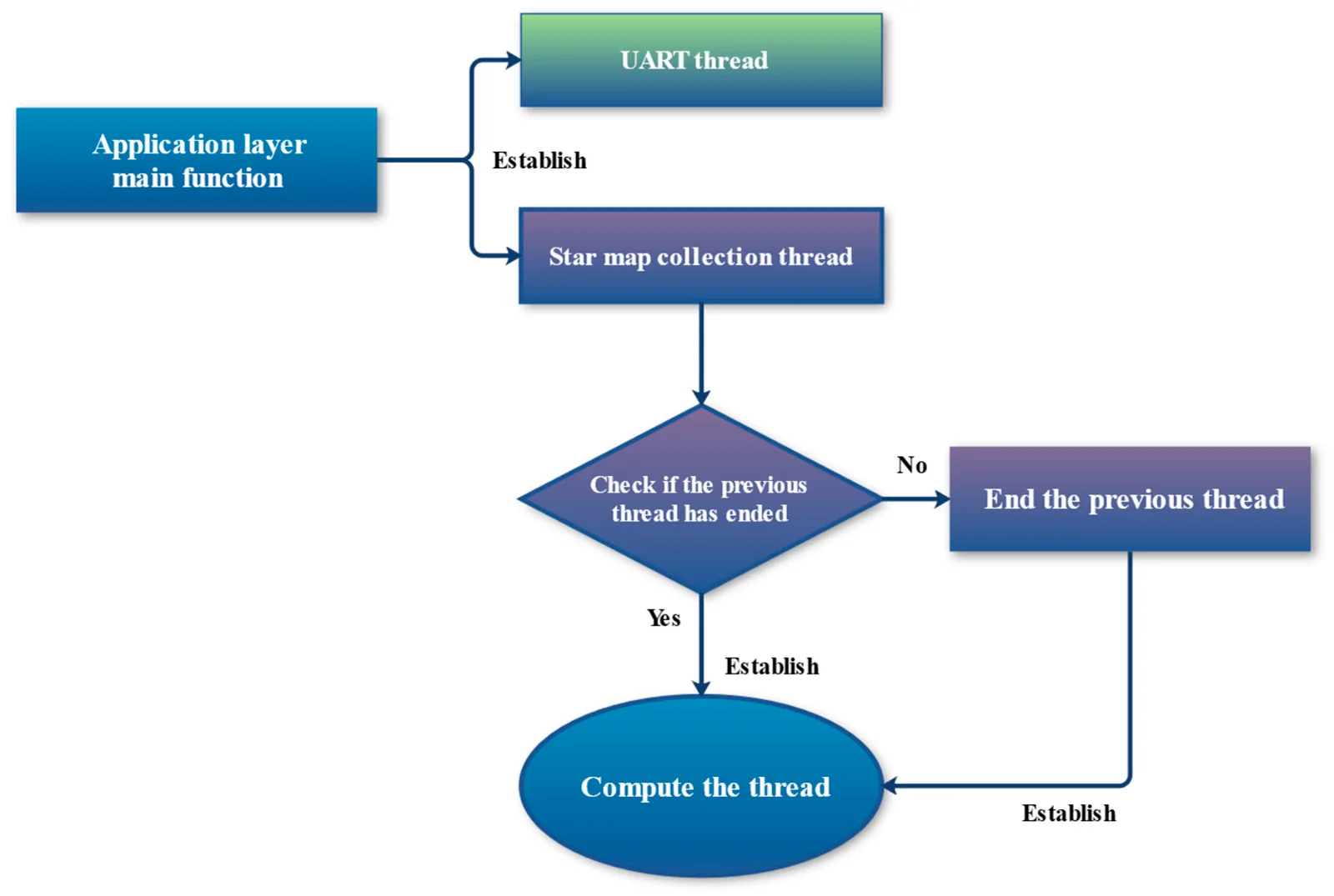
Application layer software thread of the star tracker.

Thanks to the adoption of a multi-tasking pipeline software optimization design, the nano-star tracker has ample time for star map calculations, enabling the implementation of the all-sky LIS operational mode. The key feature of this mode is that each frame of the star map undergoes an independent initial attitude recognition, eliminating the risk of tracking loss. The relationship between consecutive image frames was only used for verifying the validity of attitude calculations, resulting in higher operational efficiency and greater adaptability in space environments. Figure 5 shows the thread allocation method of the star tracker at the application layer.

Regarding the lens, to meet the low-cost requirements for commercial applications, we opted for a commercially available short-focus optical lens with a 16 mm focal length and an f/1.2 relative aperture, which was reinforced and enhanced for durability. However, commercial lenses often have significant distortion, which, if not corrected, can reduce the efficiency of star pattern recognition algorithms and directly impact the performance of the star tracker. Therefore, we employed a calibration process to measure and correct the internal parameters of the star tracker, including the lens focal length, principal point position, distortion coefficients, and sensor rotation angles [26]. By calibrating and obtaining the values of these internal parameters and incorporating them into the star tracker model, we could accurately determine the position of incoming starlight relative to the star tracker.

**Figure 6:**
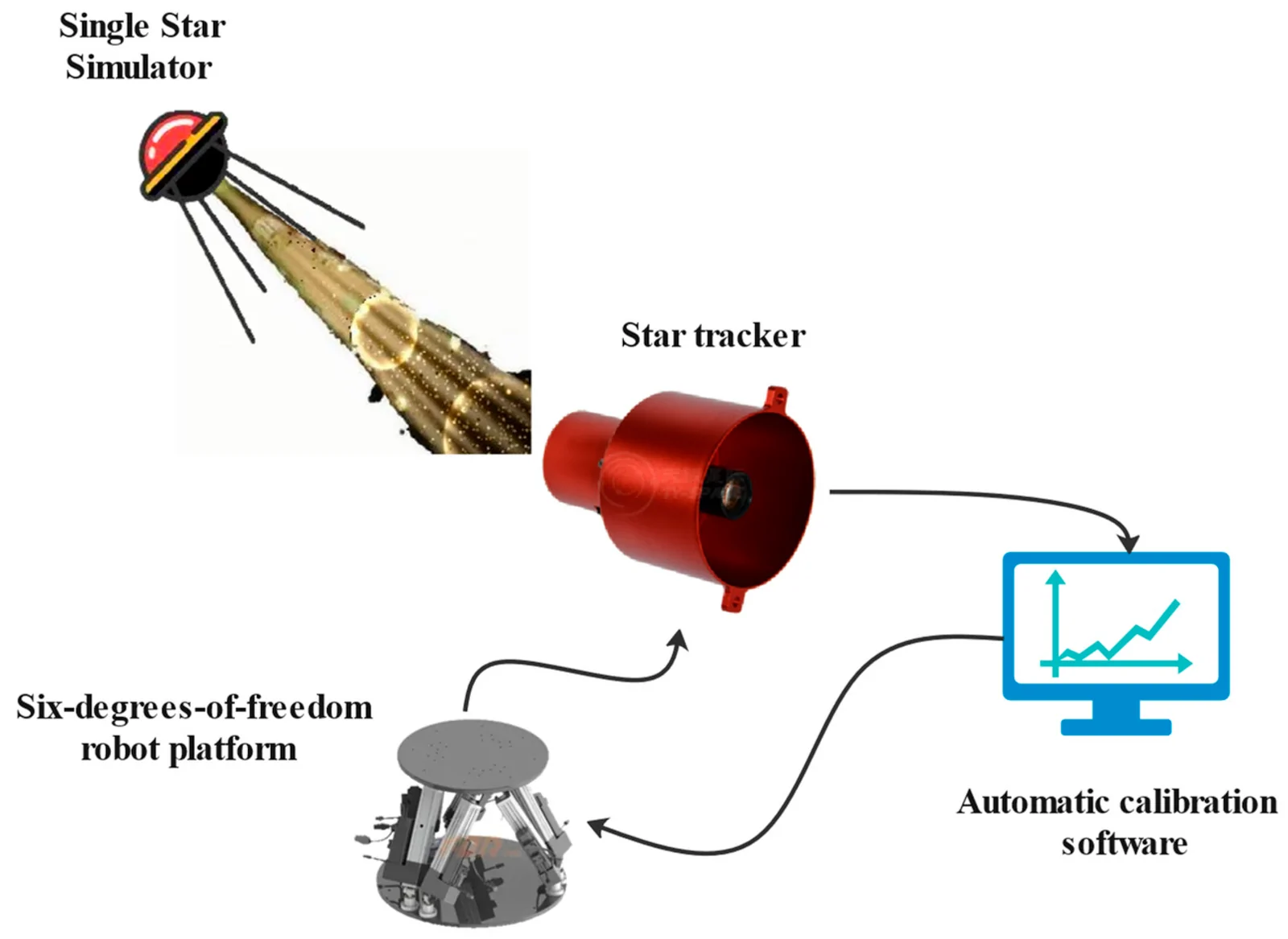
Composition of automatic calibration system for star tracker.

To facilitate this, we developed an automated calibration device for star trackers, based on a star simulator, a high-precision six-degree-of-freedom turntable, and a host computer, as shown in Figure 6. Through automated control and the acquisition of a matrix of star point dispersion spots within the sensor’s field of view, the calibration device enables the precise determination of the principal point, principal distance, and correction of lens aberrations. Traditional manual calibration devices require more than four hours to calibrate a single-star tracker, but with the automated calibration device, the time required was reduced to less than 30 min, greatly improving efficiency.

**Figure 7:**
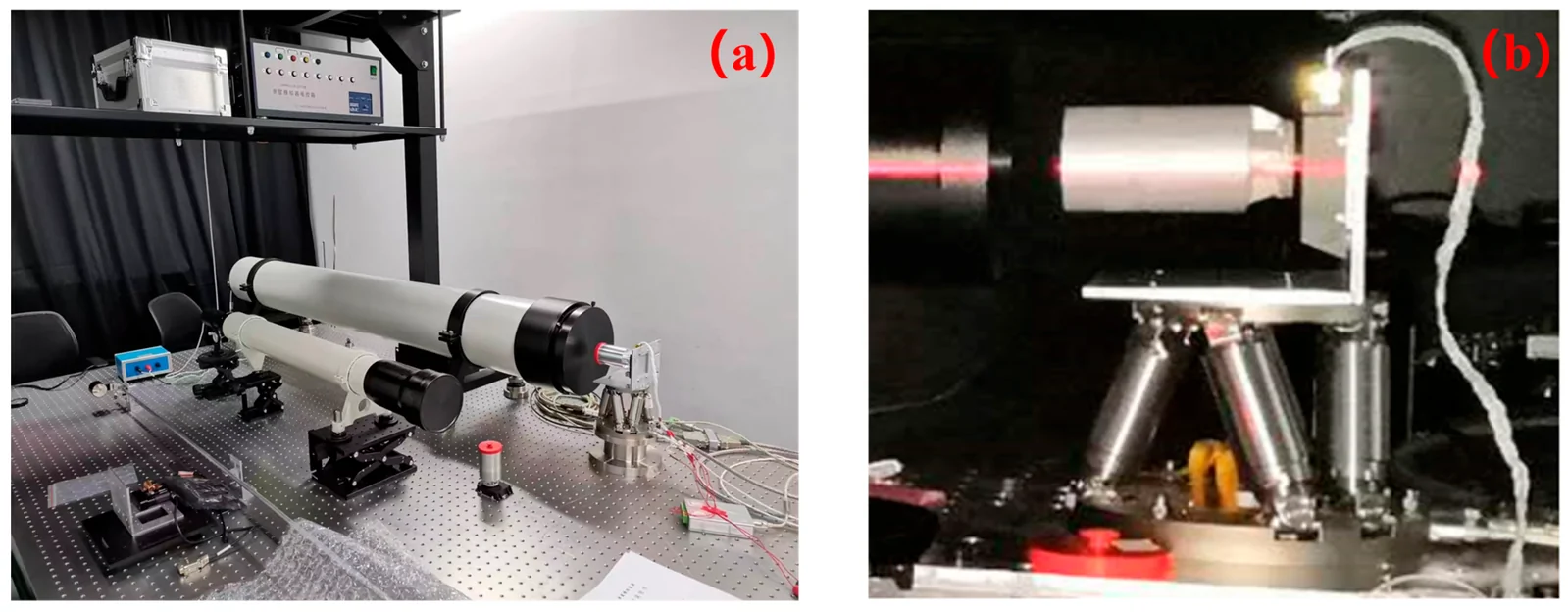
Actual picture of the star tracker calibration device: (**a**) overall view; (**b**) working state.

We simulated starlight using collimated light generated by a collimator and then rotated the star tracker using a high-precision six-degree-of-freedom (DOF) turntable to simulate starlight incidents from different directions. The turntable’s attitude was adjusted by directly inputting three parameters: the rotation angles of the XYZ axes. The star tracker was securely fixed onto the turntable’s surface. Starlight was generated using an ordinary white light source, which was diffused through frosted glass and then collimated by the collimator. Figure 7 shows the star tracker calibration device.

In this paper, the body coordinates based on the star tracker and the experimental setup were utilized, and the least squares method was employed to calculate relevant parameters, including factors such as the structure of the star tracker, the optical system, and the experimental apparatus. The body coordinates based on the star tracker, denoted as $a_{s}$ , were calculated using the inverse camera model, while the body coordinates based on the experimental setup, denoted as $a_{l}$ , were derived using the model of the experimental apparatus.

To calibrate the star tracker, a high-precision rotary table was employed to rotate the system so that the star points were imaged at different positions on the image sensor of the star tracker. These star points needed to be evenly distributed and densely cover the entire image sensor. For each imaging instance, the rotation angle of the rotary table was recorded, and the centroid of the star point image was calculated. Using the inverse camera model and the experimental setup model, ${\overset{⌢}{a}}_{s,i}$ and ${\overset{⌢}{a}}_{l,i}$ can be computed. The objective function is defined as the root mean square (RMS) of the angular difference between the two sets of body coordinates, expressed as follows [27]:

(4) $$Q=\frac{1}{N}\sum_{i=1}^{N}\arccos{\left({\overset{⌢}{a}}_{s,i}^{T}•{\overset{⌢}{a}}_{l,i}\right)}^{2},$$

where N is the total number of star point images; and ${\overset{⌢}{a}}_{s,i}$ , ${\overset{⌢}{a}}_{l,i}$ represent the unit body coordinates for the star tracker and the experimental apparatus, respectively, for the *i* star point. This problem is thus transformed into an optimization task, where the goal is to find the set of parameters that minimizes the objective function given by the aforementioned equation. The optimization process was carried out in three steps, with relevant parameter descriptions provided in Table 2 and Table 3.

1. Estimate a subset of parameters using all available data. This subset includes parameters from the camera backward model $\left(f,m_{0},n_{0}\right)$ and the experimental setup model $\left(\alpha_{1},\alpha_{2},\alpha_{3}\right)$ ;
2. Keep the parameters obtained from Step 1 unchanged and optimize the remaining parameters in the camera backward model $\left(j_{1},j_{2},\theta_{1},\theta_{2}\right)$ ;
3. Use the parameters from Steps 1 and 2 as initial values and re-optimize all parameters to obtain the final result.

**Figure 8:**
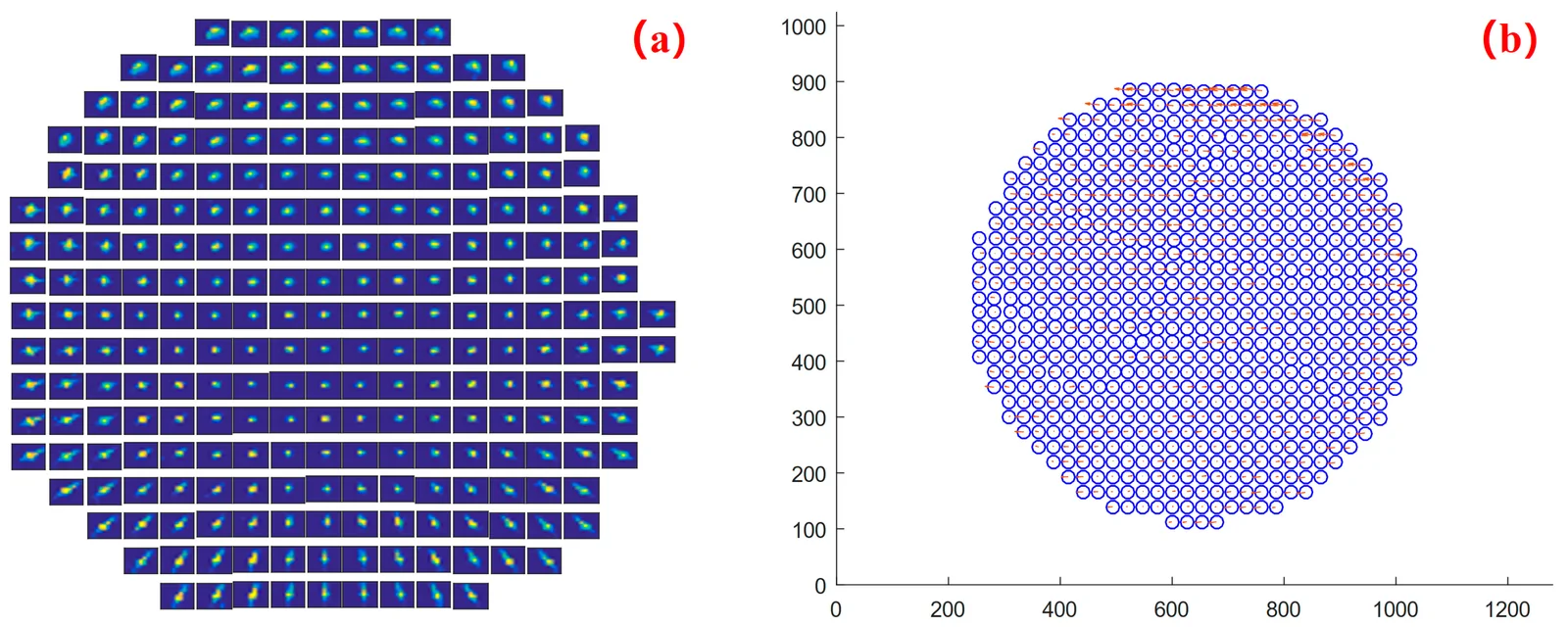
The process diagram of residual calibration: (**a**) image of the marked star points; (**b**) calibration residual diagram.

In the camera calibration procedure, star images at different positions on the surface of the image sensor were first captured, as shown in Figure 8. These star images were used for calibration, with the calibration results summarized in Table 3. The RMS of the calibration residuals is 0.0016 degrees (5.72 arcseconds), which corresponds to a 0.13-pixel error on the sensor plane. The average number of stars within the designed field of view of the star tracker was 14, and using Equation (2), the theoretical measurement accuracy can reach 1.5 arcseconds. Accurate calibration of the star tracker’s distortion is critical for ensuring the system’s measurement accuracy. Figure 8 also illustrates the calibration residuals.

During the development process, the star tracker utilizes miniaturized hardware integration technology, with optical, electronic, and structural components designed in a highly integrated manner. The specific performance indicators of the developed nanosatellite star tracker are listed in Table 4. These indicators, along with the resource consumption, meet the application requirements for precise attitude measurement of extension booms. Additionally, this nanosatellite star tracker, while using an embedded star catalog, was equipped with an automatic in-orbit star catalog correction function, which automatically compensates for factors such as stellar proper motion, light aberration, and annual parallax. The star tracker utilizing a dual-processor architecture features single event upset (SEU) resistance and programmable reconfiguration capabilities, enhancing the reliability of aerospace products. Coupled with multi-layer thermal insulation and an auxiliary thermal control system, it is well-suited for applications in the harsh conditions of space.

Compared to similar products on the international market, the star tracker we have developed is characterized by its compact size, low power consumption, and low cost. It also meets the advanced international standards in terms of technical specifications, indicating a promising application prospect. Table 5 presents the main parameters of the advanced miniature star trackers from around the world.

## 3. Ground Testing and Experiments

**Figure 9:**
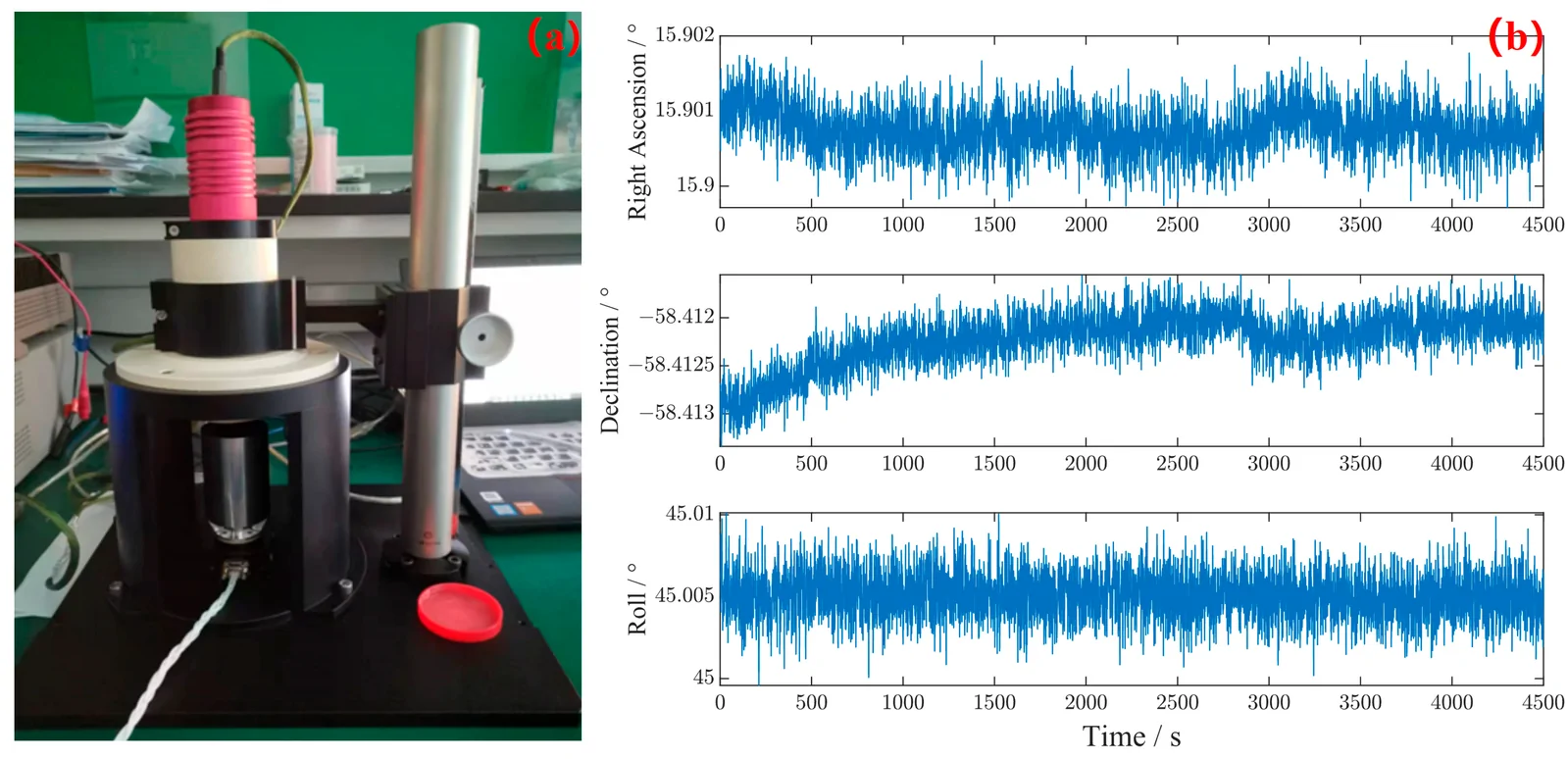
Static multi-satellite simulator experimental test diagram: (**a**) the static multi-satellite simulation test device; (**b**) the test results.

To test and ensure the in-orbit reliability of the star tracker, we conduct system-level integrated satellite testing on the ground. We first conducted tests using a static multi-star simulator in the laboratory. Its working principle is as follows: a star map, composed of fixed-point light sources, is provided, containing precise star positions and accurate angular distances between star pairs. The light generated by the simulated star points was collimated through an optical collimation system, producing parallel light rays. These rays simulate parallel light from distant stars at the entrance pupil of the star tracker’s optical system, effectively creating an optical simulation of space star maps in a laboratory setting, allowing us to replicate outdoor star observations [32]. The static multi-star simulator we used has a field of view greater than 20 degrees, with light source stability and uniformity better than 5% and a spectral range of 500 nm to 800 nm. Figure 9 presents the setup and data from an indoor experiment using the multi-star simulator. The experiment was conducted at a temperature of 25 °C, with the optical axis of the multi-star simulator aligned with the nanoscale star tracker. Over a 60-min sampling period, the star matching success rate was 100%, with pitch and yaw measurement accuracy of approximately 1.0011 arcseconds and roll measurement accuracy of about 5.1312 arcseconds. The test results demonstrate that the pointing accuracy of the nanoscale star tracker’s optical axis reaches the arcsecond level.

**Figure 10:**
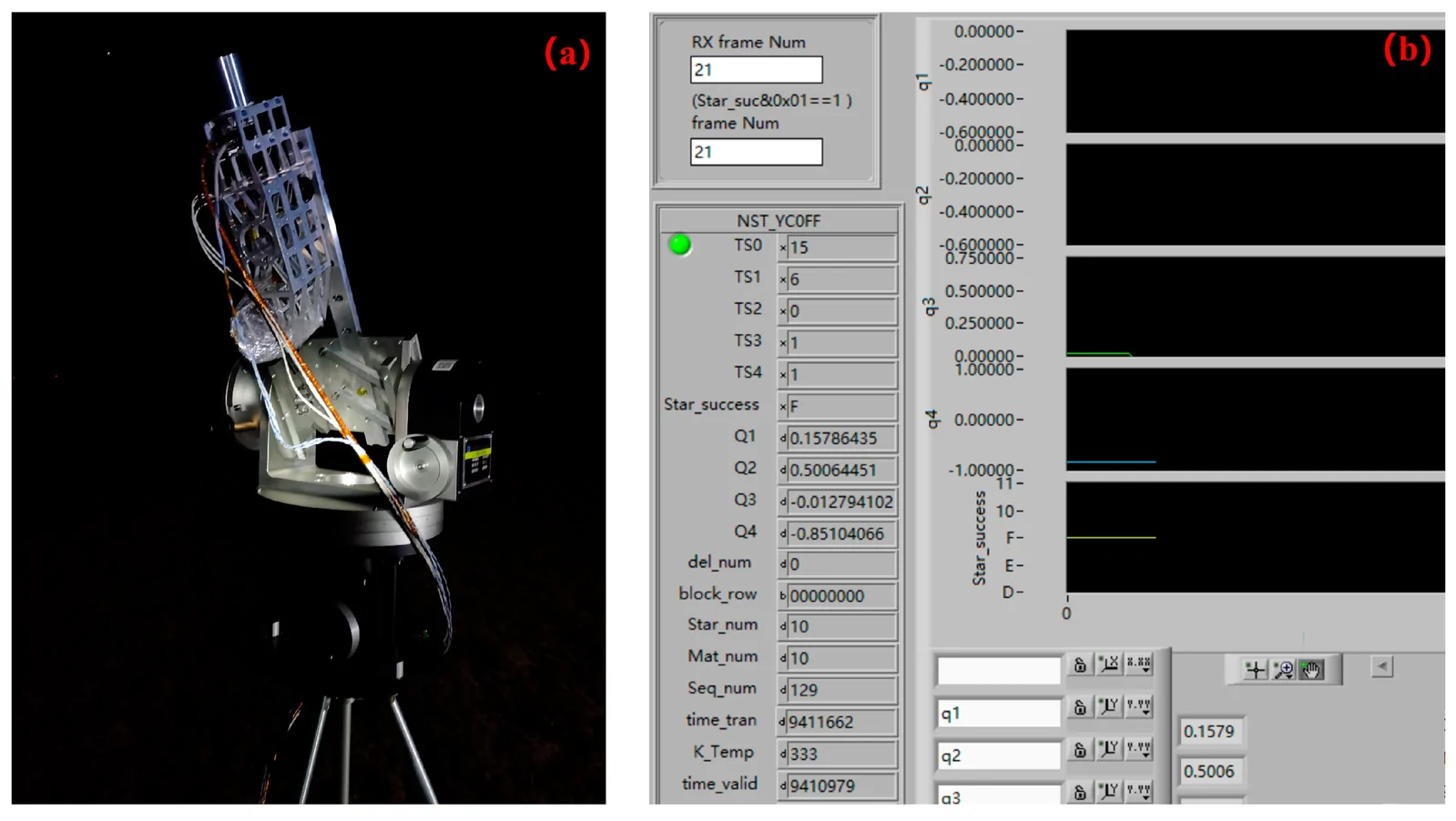
Ground test diagram: (**a**) the joint field stargazing experiment device of the probe assembly; (**b**) measurement results of the star tracker, where Q1, Q2, Q3, and Q4 represent the tracker’s output attitude quaternions.

Then, we also carried out field star observation tests and dynamic star observation tests on the nano-star tracker we developed to verify the measurement accuracy and dynamic performance of the star tracker. Figure 10 shows the field test setup and test results. This setup serves two main purposes: first, to calibrate the coordinate transformation matrix between the magnetic coordinate system of the vector magnetic field measurement device and the coordinate system of the star tracker; and second, to conduct star observation tests in three different orientations—directly facing the zenith, tilting 15° around the vertical axis, and tilting 30° around the vertical axis. In the latter two orientations, the setup was also rotated 360° around the vertical axis, and experimental validation was performed at multiple points. The sensor successfully identifies stars 100% of the time when facing the zenith. However, when tilts occur, due to atmospheric refraction and the sparsity of target stars in different sky regions, there are a few points where stars can not be identified with 100% accuracy.

Subsequently, we tested star observation images under different exposure times. The same star tracker was used to capture star maps of the same star field with varying exposure times. Table 6 presents the mean and standard deviation of these star maps.

**Figure 11:**
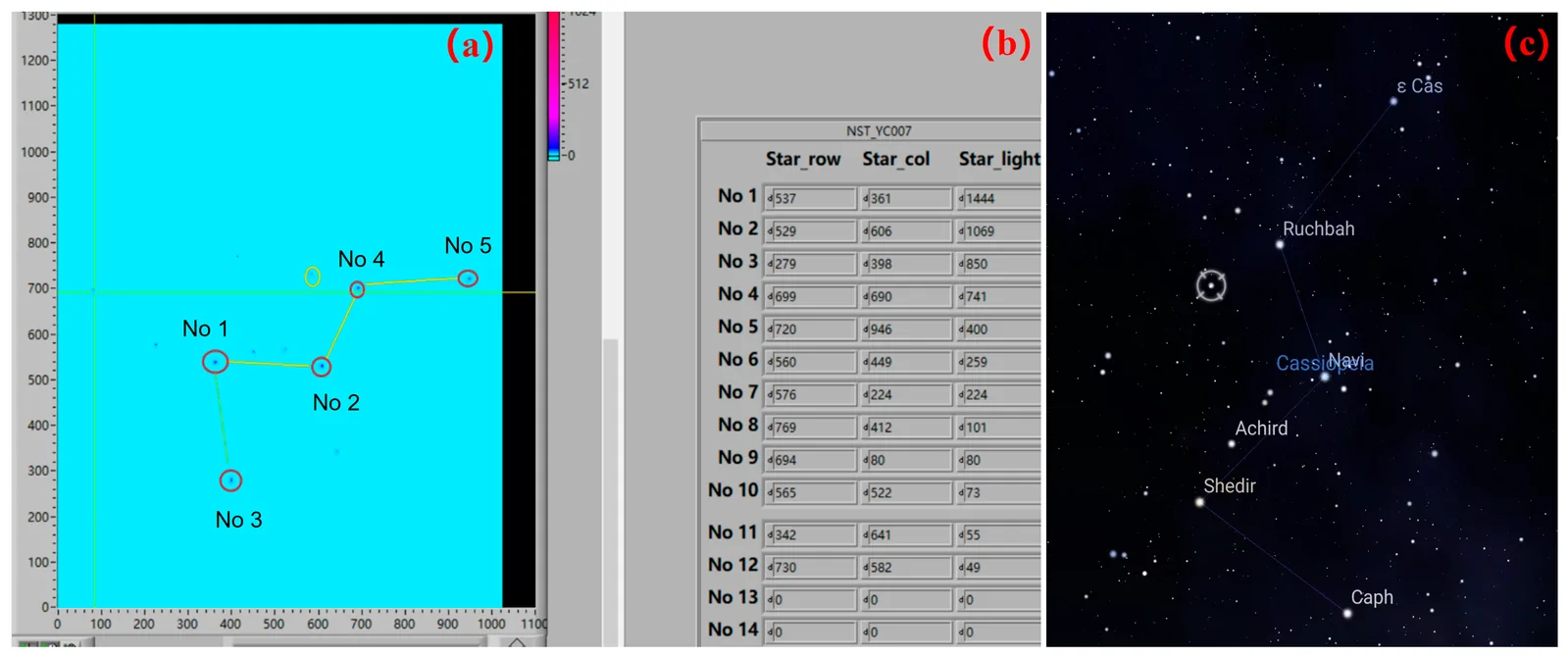
Measured star point image data of 100 ms exposure of star tracker—“Casiopeia”: (**a**) image collected by the star sensor; (**b**) the star point data analyzed in the software, which corresponds one-to-one with the identified stars; (**c**) starry sky image of the “Cassiopeia” position in the Stellarium software (v1.28).

As the exposure time increases, the mean pixel value of the images rises from 0.47939 to 1.9495, while the standard deviation rises from 2.2905 to 3.6597. Due to the preprocessing effects of the black level correction function, the data exhibit nonlinear changes but both the mean and standard deviation follow an upward trend. According to the star tracker’s star-finding algorithm, with an exposure time of 100 ms, the signal-to-noise ratio (SNR) reaches 3.538, which is sufficient to meet the star acquisition threshold. As the exposure time increases, the SNR tends to improve. Figure 11 shows the extracted star image data from the star tracker under a 100 ms exposure. Comparison with the virtual star chart software confirms that the observed “M”-shaped five bright stars correspond to the constellation Cassiopeia. The star within the yellow circle has an apparent magnitude of 4.95, which, after accounting for atmospheric extinction, was adjusted to 5.12, verifying the star tracker’s detection capability under 100 ms exposure conditions.

**Figure 12:**
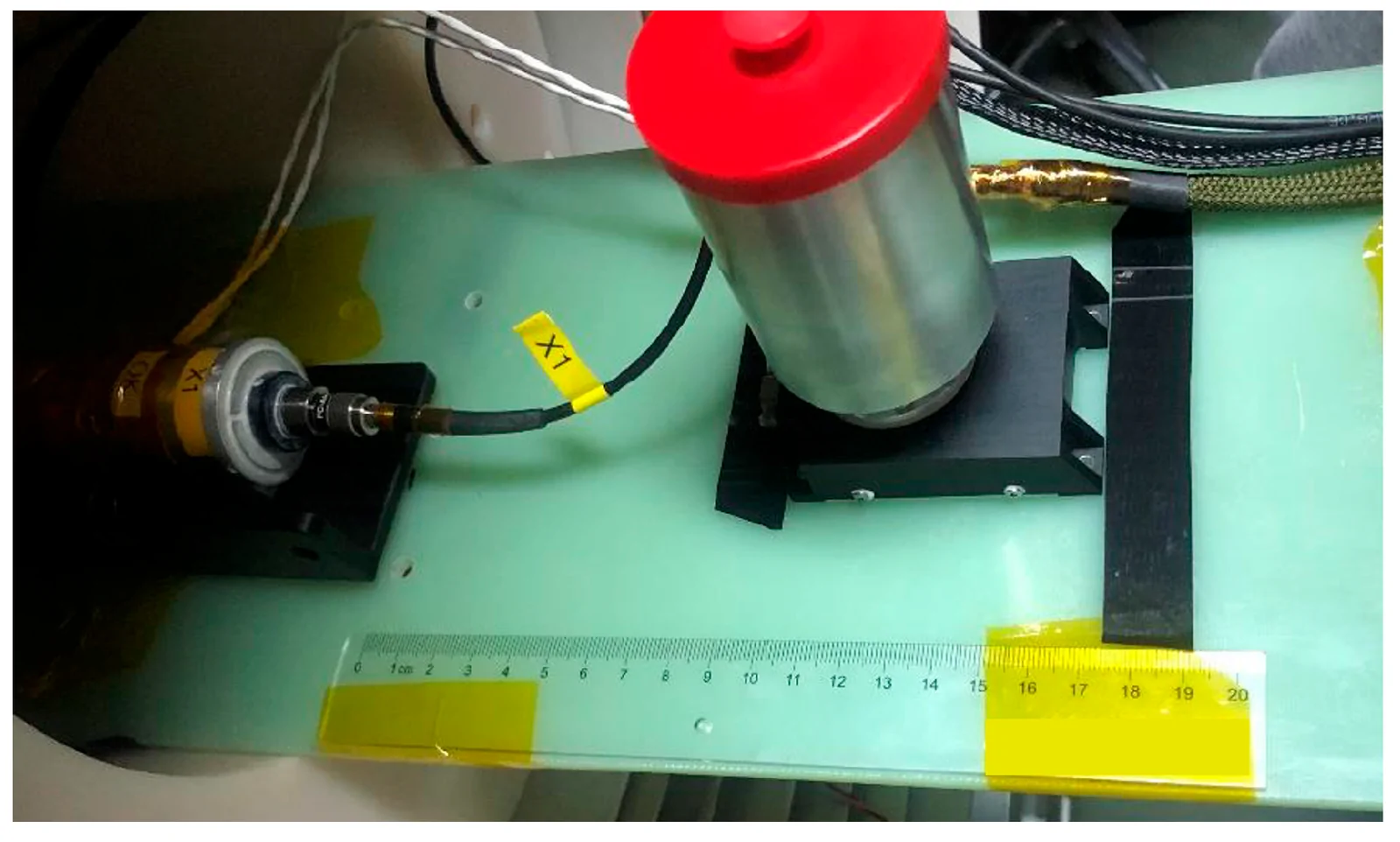
Remanence test experiment.

In response to the need for residual magnetism optimization for precise attitude measurement of the magnetometer boom by the star tracker, this paper conducts residual magnetism tests on the developed nano-type star tracker. During the residual magnetism tests, the minimum distance between the star tracker’s attitude measurement component and the magnetic field detection instrument is approximately 10 cm, as shown in Figure 12. This distance is not less than the actual installation distance of the star tracker. The measured residual magnetic signal represents the vector projection of the interference magnetic field along the total magnetic field direction. When the star tracker is powered off, the residual magnetic signal is measured as 1.5 nT, and the interference when powering the tracker on and off is also around 1.5 nT. The residual magnetism of the star tracker connector was measured at 0.7 nT. All of these measurements were taken at the designated installation distance. The test results indicate that the optimized design of the star tracker exhibits favorable residual magnetic properties, essentially meeting the usage standards. The remaining magnetism is primarily located in the connector, and by replacing with non-magnetic connectors and fasteners, the residual magnetic requirements can be better satisfied.

## 4. In-Orbit Testing and Data Discussion

**Figure 13:**
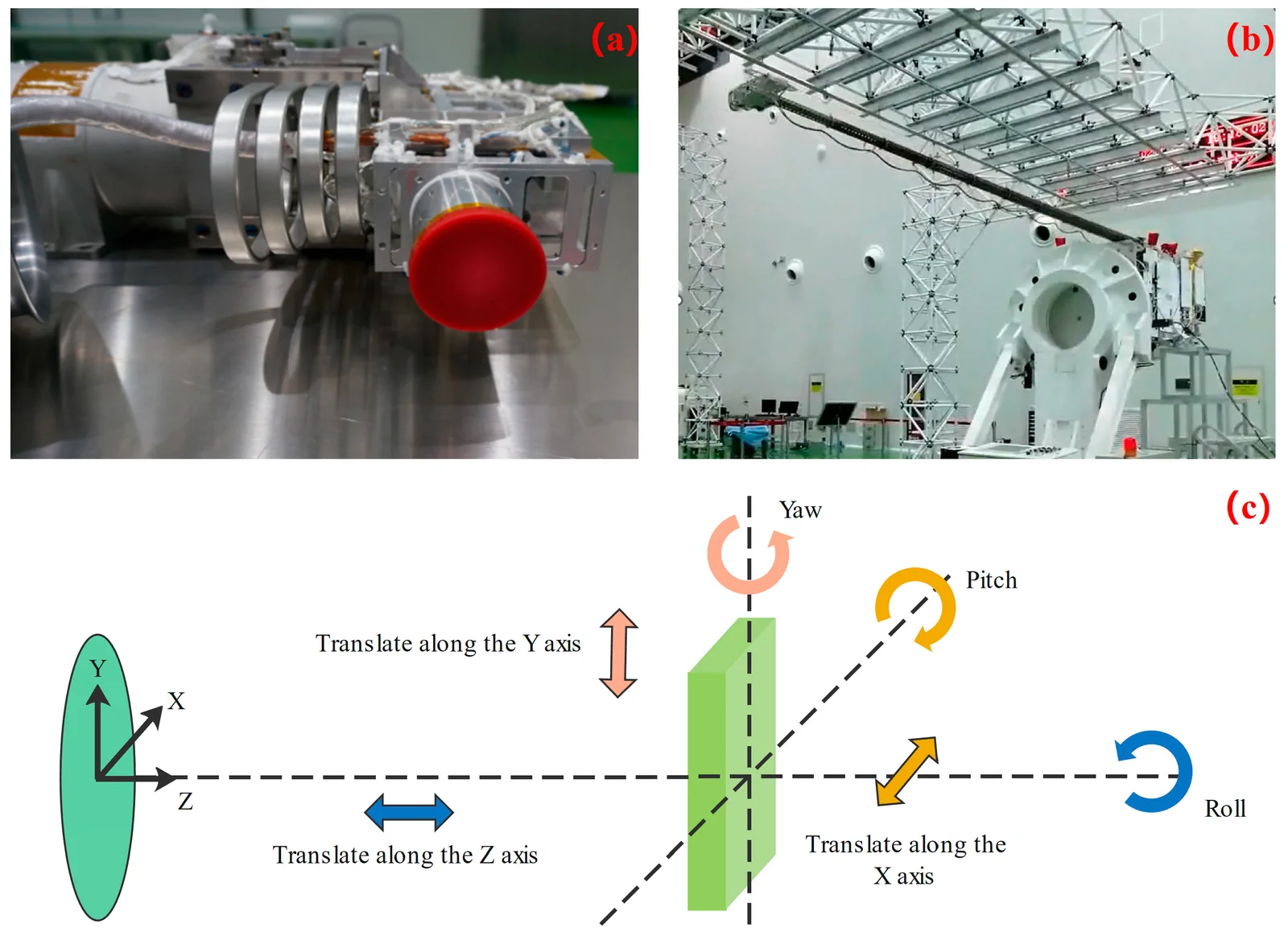
Assembly diagram of the star tracker on the Chinese Advanced Space Technology Demonstration Satellite: (**a**) the probe assembly on the extension boom, with the red light shield covering the precise attitude measurement component of the nanosatellite star tracker developed in this study; (**b**) assembly diagram of the star tracker and extension rod structure on the entire satellite; (**c**) the coordinate system relationships of the satellite’s extension boom, where the satellite platform’s boom base is defined as the *XY* plane and the boom’s extension direction is defined as the *Z*-axis.

To thoroughly validate the capability of the nanosatellite star tracker in the precise attitude measurement system of space extension booms, we apply the attitude measurement component of the nanosatellite star tracker to the 5-m magnetometer boom of the Chinese Advanced Space Technology Demonstration Satellite developed by the Innovation Academy for Microsatellites of the Chinese Academy of Sciences. Figure 13 shows the assembly diagram of the star tracker on the Chinese Advanced Space Technology Demonstration Satellite. During in-orbit operations, the attitude of the satellite’s boom can exhibit variations across six degrees of freedom, including translational movements along the *X*, *Y*, and *Z* axes as well as rotational angle changes around the *X*, *Y*, and *Z* axes. The nanosatellite star tracker’s optical axis is oriented perpendicular to the *Z*-axis direction of the boom. By leveraging the tracker’s measurement accuracy in the optical axis and roll directions, we can achieve angular measurements of the boom’s rotation around the *Y* and *Z* axes with an accuracy of 3–5 arcseconds and measurements around the *X*-axis with an accuracy better than 50 arcseconds.

**Figure 14:**
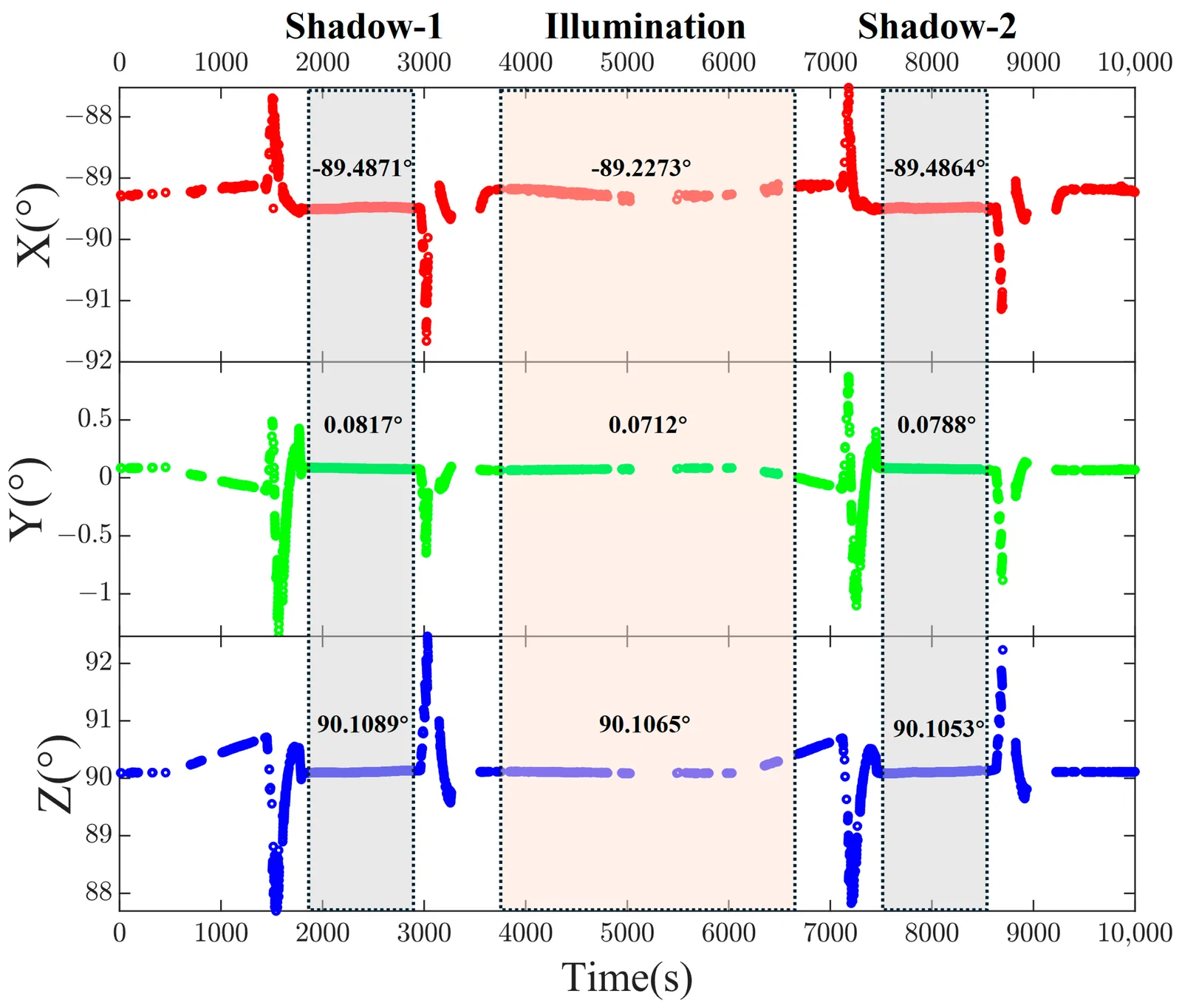
Conversion Euler angles from NST system by the star tracker to satellite system, the time range of the data in the figure is UTC: 13 February 2023 9:09:10 to 12:05:50.

The Space Advanced Technology demonstration satellite has already been successfully launched and has transmitted data collected by the star tracker. We have processed the relevant information and selected data from specific time periods, focusing on the satellite’s attitude and the star tracker data from the boom during different conditions: within the shadow region, the illuminated region, and the transition from shadow to light. Figure 14 presents the calculated Euler angles for the transformation from the NST (nanosatellite) coordinate system to the satellite’s body coordinate system.

The Euler angles describing the transformation from the NST (nanosatellite) coordinate system to the satellite body coordinate system illustrate the attitude relationship between the extension boom and the satellite body. In the shadow region, the Euler angles remain relatively stable, with the sequence holding steady around [−89.49°, 0.08°, 90.11°] for approximately 1200 s in the first shadow period, with a standard deviation of 15″ (1σ). This indicates that the extension boom’s attitude is stable in the absence of illumination. In the illuminated region, which lasts about 3800 s, the Euler angles stabilize around [−89.23°, 0.07°, 90.11°], with a standard deviation of 30′ (1σ). This slight increase in variability suggests that the extension boom experiences some changes due to temperature variations caused by solar illumination, but overall, it maintains a stable attitude.

Comparing the X-Y-Z rotation sequences, the difference in the average values between the first and second shadow regions is minimal, at [0.0007°, 0.0029°, 0.0036°]. In contrast, the difference between the first shadow region and the illuminated region is more pronounced, at [0.2598°, 0.0105°, 0.0024°], which can be attributed to thermal deformation of the extension boom due to solar heating.

In the transition zone between the shadow and illuminated regions, the Euler angles exhibit significant changes. The Euler angles in the *X* and *Z* directions fluctuate considerably, with the pitch and roll angles of the extension boom relative to the satellite body varying by up to ±2°. This is likely due to variations in the star tracker’s optical axis orientation as the satellite undergoes orbit correction and attitude adjustments. The Euler angle in the Y direction shows smaller fluctuations, generally remaining within ±1°, indicating that the yaw angle remained relatively steady. Such changes do not affect the relative position between the star tracker and the extension boom, and therefore have no impact on attitude determination.

**Figure 15:**
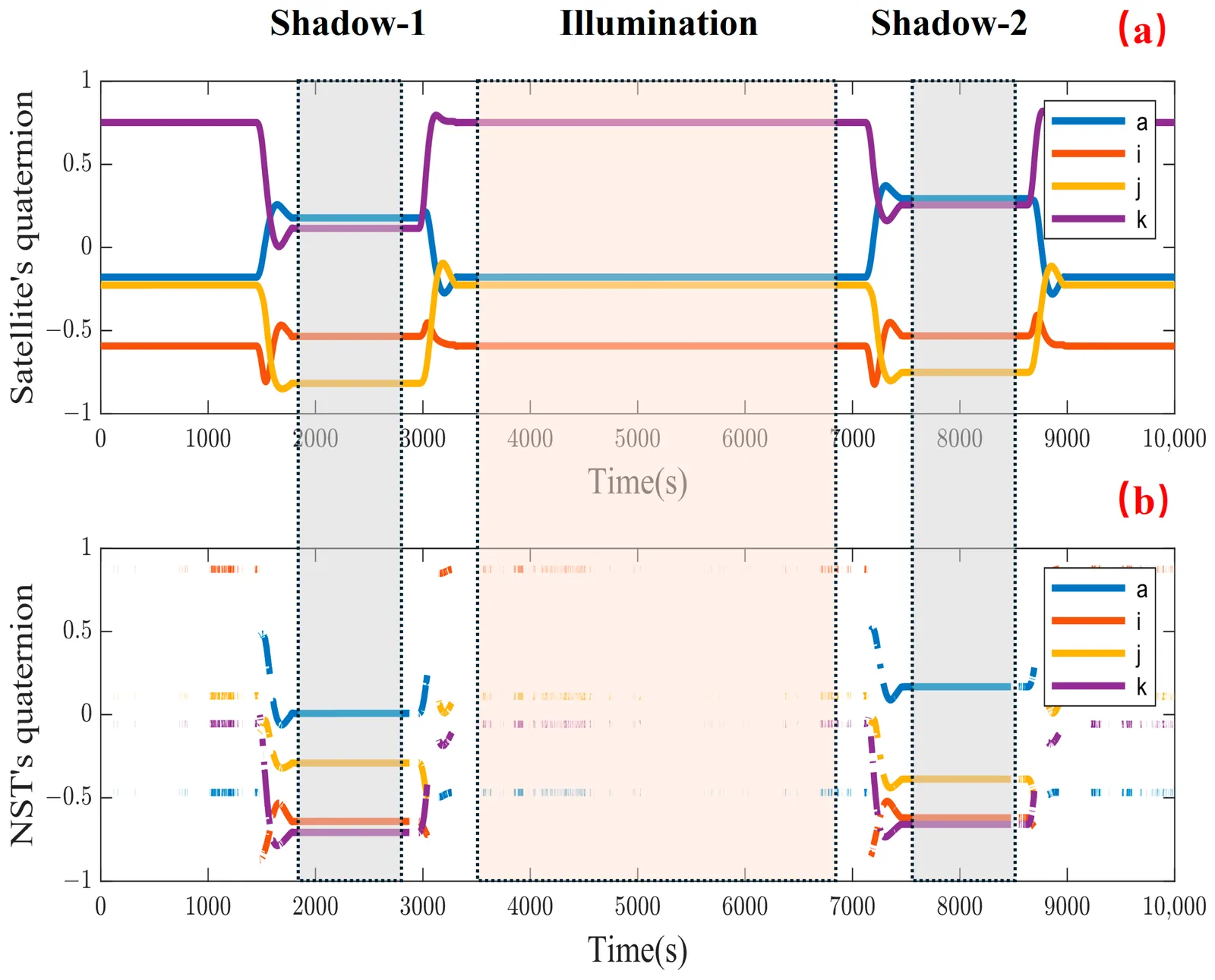
Quaternions collected by the star tracker: (**a**) data sourced from the star tracker mounted on the satellite body; (**b**) data sourced from the star tracker on the extension boom. The time range of the data in the figure is UTC: 13 February 2023 9:09:10 to 12:05:50.

Figure 15 illustrates the calculated quaternion results, where the satellite broadcast attitude quaternion data are sourced from the star tracker mounted on the satellite body. The variations in these quaternions primarily result from orbital adjustments and attitude corrections during different mission phases, such as solar observation, transitions between daylight and night (shadow–light–shadow transitions), and other key time points. The satellite body’s attitude quaternions exhibit clear periodicity and stability, with consistent patterns across the different components. Correspondingly, the NST attitude quaternions are derived from the star tracker installed on the deployable extension boom, providing attitude data for the boom at each moment. The data collected in the shadow region are relatively complete, while the data under illuminated conditions appear more scattered. This discrepancy is primarily due to the use of a commercial lens for the star tracker in this mission, which has significant limitations in shielding stray light. Ground laboratory tests have shown that even with a solar suppression angle greater than 35°, stray light still enters the star tracker. Consequently, the star tracker’s normal operation is compromised in illuminated environments, recognizing only a few stars for attitude determination, which impacts data accuracy. However, this problem can be solved through subsequent calibration algorithms and will not affect the final attitude solution. The quaternion variations in the extension boom follow the changes in the satellite’s orbit and attitude corrections, and are further influenced by environmental factors such as temperature and gravitational fields.

Analysis of the in-orbit data reveals that the primary attitude changes in the 5-m-long boom during space operations occur during transitions in and out of the shadow region. The main cause of these changes is the satellite’s orbital adjustments and attitude corrections, leading to variations in the boom’s attitude; however, once these processes conclude, the boom’s attitude quickly stabilizes, with the total variation remaining within ±2°. Additionally, within the shadow region, the boom’s attitude shows minimal changes, particularly in the yaw and roll angles, which remain very stable. In contrast, in the illuminated region, the attitude angles across all three dimensions exhibit greater fluctuations compared to those in the shadow region, indicating that temperature conditions do have an impact on the extension boom’s attitude.

## 5. Conclusions

To meet the precise attitude measurement requirements of deployable space structures, this paper first reviewed the current research status of nanoscale star trackers internationally, outlining the relevant technical specifications and requirements. Next, using miniaturization and hardware integration techniques, the optical, electronic, and structural components of the star tracker were designed with a high degree of integration. A prototype nanoscale star tracker, suitable for measuring the attitude of deployable arms, was successfully developed. The star tracker’s application software underwent multi-task pipeline optimization, achieving an attitude update rate of better than 5 Hz in the LIS operating mode. The star tracker also underwent dark field calibration, residual magnetism testing, reliability testing, field star observation tests, and joint calibration with the magnetometer probe on the extension boom. After verifying the relevant technologies—which are self-designed and deployed in the attitude measurement system of the extension boom on the Chinese Advanced Space Technology Demonstration Satellite—the star tracker was found to enable simultaneous in-orbit attitude measurement verification, providing in-orbit attitude data for a 5-m-long boom. The results reveal that in the shadow zone, the Euler angles stabilized around [−89.49°, 0.08°, 90.11°], with a standard deviation of 15″ (1σ); in the illuminated zone, the Euler angles stabilized around [−89.23°, 0.07°, 90.11°], with a standard deviation of 30′ (1σ); and in the transition zone, the maximum angle fluctuation was ±2°. Analysis of the results indicates they are within a reasonable range. Although the use of low-cost, commercial star tracker lenses led to some data loss, further data processing is expected to improve the results. Additionally, these findings provide valuable data support for further improvements to the star tracker in the future.

## Figures and Tables

**Table 1:** Comparison of precision attitude measurement techniques for space extension booms.

Sensor Type	Advantages	Disadvantages
GPS [14]	3D absolute positioning	Max accuracy 0.3 mm
Inertial Measurement Unit (IMU) [15]	Autonomous, interference-resistant, real-time	Drift over time; requires correction
Beacon light measurement unit [16]	Multi-point position resolution	Complex data processing
Position-sensitive measurement unit [12,17]	High frame rate, simple, high precision	No multi-point resolution
Infrared horizon sensor [18]	Earth vector measurement, mature tech	Low accuracy; 2 DoF measurement
Star tracker [2,3,4,5]	3D absolute angle, arcsecond precision, autonomous	High cost, large size, strict magnet control

**Table 2:** Model parameters and their meanings.

Parameter	Symbol	Meaning
Rotation Angle of the Turntable	$\theta_{1}$ , $\theta_{2}$ , $\theta_{3}$	The angle by which the high-precision turntable rotates in response to user commands
Rotation Angle of the Star Tracker	$\alpha_{1}$ , $\alpha_{2}$ , $\alpha_{3}$	The tilt angle of the lens and sensor assembly of the star tracker

**Table 3:** Parameters obtained from model calibration.

Parameter	Symbol	Unit	Value
Rotation Angle of the Star Tracker	$\alpha_{1}$ , $\alpha_{2}$ , $\alpha_{3}$	radians	0.0011, 0.0033, 0.0018
Focal Length	f	mm	15.9778
Principal Point	$m_{0},n_{0}$	pixels	620.9684, 583.8224
Radial Distortion Coefficient	$j_{1},j_{2}$	/	−0.2581, −1.8695
*Y*-axis Size Factor	$c_{s}$	/	0.9995

**Table 4:** Performance index of the star tracker.

Technical Specification	Measured Value
Field of View (FOV)	Circular 16°
Attitude Measurement Accuracy	Pitch and Yaw: 3–5 arcseconds (1σ) Roll: better than 50 arcseconds (1σ)
Weight	150 g
Envelope Dimensions	106 × 77 × 50 mm
Default Data Update Rate	5 Hz
Operating Mode	Full-sphere LIS (Lost In Sight) mode
Sun Suppression Angle	35°
Power Supply Voltage	Normal operation from +4.5 V to +5.5 V
Average Power Consumption	0.85 W at a single 5 V power supply
Operating Temperature Range	−40 °C to +45 °C
Storage Temperature Range	−40 °C to +80 °C

**Table 5:** Parameters of the leading international miniature star trackers.

Development Unit	Model	Weight/g	Volume/mm^3^	Update Rate/Hz	Power Consumption/W	Pointing Accuracy (1σ)
Ryerson/Sinclair Interplanetary [22]	ST16	185	62 × 56 × 68	2	Average 0.5, Peak 1	5″
BST [28]	ST200-μD	106	50 × 50 × 83	5	0.67	10″
BST [29]	ST400D	350	74 × 74 × 146	5	0.75	5″
Jena-Optronik [30]	ASTRO-CL	300	60 × 60 × 175	5–8	1	2″
SODERN [31]	Auriga	520	91 × 117 × 25	8–10	0.8–1.1	3″

**Table 6:** Summary of the parameters required for the laboratory model.

Exposure Time (ms)	Image Mean (LSB)	Image Variance (LSB)
100	0.47939	2.2905
200	1.5293	2.6941
300	1.6978	2.7391
400	1.8173	3.3684
500	1.9495	3.6597

## Data Availability

Due to the sensitive nature of space science data, the data presented in this study are available from the corresponding author upon request.
